# Dendritic cells under the control of the preimplantation embryo secretome: an in vitro study

**DOI:** 10.1186/s12958-024-01319-2

**Published:** 2024-11-23

**Authors:** Christiana Kyvelidou, Sofia Haselrieder, Maria von Gierke, Johanna M. Gostner, Wolfgang Biasio, Barbara Wirleitner, Christine Heufler, Bettina Toth, Susanne Hofer-Tollinger

**Affiliations:** 1 Department of Gynaecological Endocrinology and Reproductive Medicine, Medical University of Innsbruck, Anichstraße 35, Innsbruck, 6020 Austria; 2 Institute of Medical Biochemistry, Biochemical Immunotoxicology Group, Biocenter, Medical University of Innsbruck, Innsbruck, Austria; 3 Next Fertility IVF Prof. Zech, Bregenz GmbH, Bregenz, Austria; 4 Department of Dermatology, Venereology & Allergology, Medical University of Innsbruck, Innsbruck, Austria

**Keywords:** Preimplantation embryo, Blastocyst, Embryo spent media, Dendritic cells, Maternal tolerance

## Abstract

**Objective:**

To study the crosstalk between maternal immune cells and the developing embryo by investigating the immunogenic properties of human blastocyst spent media (SM) on dendritic cells.

**Methods:**

In this prospective multicenter experimental study, human preimplantation embryo spent media were collected after blastocyst formation, grouped based on successful or unsuccessful implantation, and analyzed by protein array or used to stimulate monocyte derived dendritic cells (moDC). The immunomodulatory properties of SM on moDC were investigated by analyzing changes in phenotype, cytokine secretion, indoleamine 2,3-dioxygenase (IDO) activity, and ability to activate T cells.

**Results:**

A plethora of cytokines and growth factors secreted from preimplantation embryos was detected. Exposure to embryo SM altered the phenotype of moDC in a manner dependent on the implantation outcome. Specifically, SM from non-implanted embryos increased the expression of co-stimulatory molecules and activation markers on moDC. Furthermore, SM treated dendritic cells secreted low levels of cytokines and growth factors and were able to stimulate naïve T cells. Activation of IDO was decreased in moDC after stimulation with SM.

**Conclusions:**

Our findings show that human preimplantation embryos secrete an abundance of molecules with the ability to significantly affect and even regulate immune cells in their environment.

**Supplementary Information:**

The online version contains supplementary material available at 10.1186/s12958-024-01319-2.

## Background

Maternal immune tolerance is pivotal for the successful development of the semi-allogeneic fetus, involving intricate cell-cell communication processes. Maternal cells secrete various signaling molecules that prepare and maintain the optimal environment for implantation and pregnancy [1–3]. Simultaneously, the embryo itself secretes signaling molecules to modulate its surroundings, even before implantation. Despite the significance of this process, the precise mechanisms governing tolerance at the embryo-maternal interface remain largely elusive.

In spontaneous conceptions, the live birth rate for women under 35 years old is 25-30% and the pregnancy rate is 30% on the first attempt [4], indicating that a substantial number of oocytes or embryos fail to fertilize or implant respectively [5]. Similarly, assisted reproductive technology (ART) yields a pregnancy rate of approximately 35%, depending on different risk factors (i.e., maternal age, congenital or anatomical abnormalities, or infections) [6]. Although recent studies have reported increased implantation rates for women under 35 years [7–9], the need to understand the mechanisms involved in successful implantation remains.

Prior to implantation, the uterus undergoes necessary preparations characterized by endometrial thickening, vascular transformation, and secretion changes. These are accompanied by immunological changes facilitated by factors like leukemia inhibitory factor (LIF) and interleukin 1β (IL 1β), secreted by maternal cells [10, 11]. Uterine natural killer (NK) cells and dendritic cells (DC) play crucial roles in decidual transformation by providing signals such as transforming growth factor β1 (TGF-β1) and soluble Fms-like tyrosine kinase-1 (sFlt1) [12, 13].

Human implantation initially requires a proinflammatory environment, characterized by the presence of various proinflammatory signals and cytokines. The “implantation window”, typically defined as days 19–24 of a 28-day menstrual cycle, is the period when the endometrium is optimally receptive for the implantation of a free-lying blastocyst [14]. During this timeframe, prostaglandin E2 (PGE2), tumor necrosis factor α (TNFα), and IL1β are released in the uterus, priming the endometrium for the invading embryo [15]. Recent studies revealed reduced post-implantation levels of T-cell immunoglobulin mucin-3 (Tim-3) and programmed death-1 (PD-1), molecules involved in anti-inflammatory pathways [16]. However, as implantation progresses, the embryo’s microenvironment shifts to an anti-inflammatory state to prevent rejection and support fetal growth [17].

DC are vital regulators of the immune system, orchestrating both pro-inflammatory and tolerant/anti-inflammatory immune responses by interacting with and stimulating other immune cells either directly or through cytokine and chemokine secretion. Immunogenic and tolerogenic DC differ in their function, e.g., their ability to induce different T cell subsets, and their characteristics, e.g., the expression of co-stimulatory molecules or surface markers, the production of different signaling molecules, and the expression of the tryptophan catabolizing enzyme indoleamine 2,3-dioxygenase 1 (IDO-1). IDO-1 plays a crucial role in regulating immune responses and is expressed at the embryo-maternal interface suppressing maternal T cell responses to fetal alloantigens, while directly activating resting Treg [18].

DC are present in the follicular fluid, the ovary, and the decidua, where they can influence different aspects of the female reproductive system. For instance, in the ovaries they participate in the expansion of the cumulus-oocyte complex, the release of the mature oocyte, and the formation of the corpus luteum [19], while in the decidua their interactions with Treg impact the invasion of the extravillous trophoblast [20].

Although comprising only 1–2% of leukocytes in the endometrium, DC play a crucial role there exhibiting distinct immature and mature phenotypes [21]. Specifically, they can be characterized as immature non-activated (CD209+), immature activated (DEC205+), and mature activated (CD83+) [22, 23]. Most of the decidual DC in early pregnancy are found to be immature and their presence has been associated with the establishment of healthy pregnancies [24, 25]. However, their scarcity has limited research on DC and implantation.

This study aims to investigate whether the spent media (SM) from in vitro fertilized blastocysts can affect the maturation and differentiation of immature monocyte-derived dendritic cells (moDC). By exploring the impact of the embryo’s secretome on DC, we aim to gain insights into the mechanisms governing successful implantation and pregnancy.

## Methods

### Participants

Prospective participants undergoing an ART cycle were recruited. Inclusion criteria involved maternal age (> 18 and ≤ 41 years). Exclusion criteria involved known immunological disorders (e.g., antiphospholipid syndrome (APLS), multiple sclerosis, inflammatory rheumatic diseases) and active or chronic infections. The study was approved by the ethics committee of the Medical University of Innsbruck, Austria (EK Nr AN2014-0312 343/4.6) and the ethics committee of “Land Vorarlberg” (EK-2-9/2020). Signed informed consent was obtained from all participants involved in the study.

### Sample collection

Human preimplantation embryos were cultured in 23 µl of CSCM-NX (90167, FUJIFILM Irvine Scientific) supplemented with 10% SSS (99193, FUJIFILM Irvine Scientific) until the blastocyst stage in all the experiments except for the custom human Quantibody array (RayBiotech). In this case, the embryos were cultured in 23 µl of global medium (LGGG-100, CooperSurgical) supplemented with human serum albumin (GHSA-125, CooperSurgical) until the blastocyst stage. SM were collected on day 5 or day 6 depending on when the blastocyst was formed and subsequently removed either for cryopreservation or for a planned embryo transfer. No blastocyst was left in culture for a prolonged period after its formation. Each embryo transfer was conducted using ultrasound imaging to ensure precise catheter placement. Visualization of the catheter during insertion enabled alignment with the uterine curvature, facilitating optimal deposition of the embryos within the endometrial cavity via a semiautomatic dispenser.

After transfer or cryopreservation of the embryo, 23 µl of SM were collected from single embryo cultures, snap-frozen in liquid nitrogen, and stored at -80 °C until further analyses. Only SM from high-quality embryos (grade AA, AB, BA, or BB), graded as per Gardner et al., were used [26, 27]. Cultured media untouched by any embryo were used as blanks/negative controls in all experiments in a manner parallel to that of embryo SM.

After fresh or cryopreserved embryo transfers, each corresponding stored SM sample was categorized as pregnant (prSM; positive hCG in peripheral blood 16 days post embryo transfer and detection of fetal heartbeat via ultrasound around the 7th week of pregnancy) or non-pregnant (npSM; negative hCG in peripheral blood 16 days post embryo transfer). SM samples collected from embryos resulting in biochemical pregnancies (positive hCG in peripheral blood 16 days post embryo transfer, followed by a negative hCG or absence of an identifiable pregnancy on ultrasound examination) were excluded from this study and were not used in any of the experiments. No preimplantation genetic testing was carried out on the embryos included in the study. In accordance with the regulations of Austria, genetic screening is only performed when there are specific pathologies or indications that necessitate it and is not part of the routine ART procedure.

### Generation and culture of monocyte-derived dendritic cells

Leucocyte reduction system chambers (LRSC) were obtained from anonymous healthy female donors through the Central Institute for Blood Transfusion and Immunology (Medical University of Innsbruck, Innsbruck, Austria) according to the approval of the ethics committee of the Medical University Innsbruck (1265/2019). PBMC were isolated by density gradient (7811, Stemcell Technologies) from LRSC. CD14 + monocytes were further acquired through negative selection by magnetic sorting (130-096-537, Miltenyi) and purity was assessed by flow cytometry > 95%. 5 × 10^4^ monocytes were seeded in 96-well flat bottom plates in 200 µl RPMI 1640 (BE12-167 F, Lonza) supplemented with 10% FBS (10082147, Gibco), 1% Glutamax (35050038, Fisher Scientific), 1% HEPES 1 M (H0887, Sigma) and 50 µg/ml gentamycin (15750-037, Fisher Scientific). On day 0, monocytes were stimulated with 20 U/ml IL4 (130-093-91, Miltenyi) and 800 U/ml GM-CSF (Leukine sargamostim, SANOFI). On day 2, they were re-stimulated with 20 U/ml IL4 and 1600 U/ml GM-CSF. On day 5 the cells had fully differentiated in immature moDC, as determined by > 95% expression of HLA-DR (human leucocyte antigen DR). MoDC were then treated according to the different experimental groups. For the prSM-moDC group, 20 µl of embryo prSM were added. Similarly, for the npSM-moDC group, 20 µl of embryo npSM were added. For the control group of immature moDC, 20 µl of embryo culture medium (untouched by any embryo) were added. Finally, for the control group of the mature moDC, 20 µl of embryo culture medium (untouched by any embryo) were added and the cells were further stimulated with a maturation cocktail. The maturation cocktail consisted of 10 ng/ml TNFα (300–01 A, Peprotech), 2 ng/ml IL-1b (200-01B, PeproTech), 1000 U/ml IL-6 (200-06, PeproTech), and 1 µg/ml prostaglandin E2 (P5640-1MG, Sigma). In both immature and mature moDC, 20 µl of CSCM-NXC supplemented with 10% SSS were added. MoDC were cultured for 48 h. On day 7, all samples were collected and centrifuged at 300 g for 6 min. Cell pellets and supernatants were used for further analyses.

### Generation and culture of CD4+ T naïve cells

CD4 + naïve T cells were acquired from PBMC (as described above in Generation and culture of monocyte-derived dendritic cells) through negative selection by magnetic sorting (130-094-131, Miltenyi), and purity of the CD4 + CD45RA + CD45RO- naïve T cells was found > 95%. 10^5^ CD4 + naïve T cells were seeded in 96-well flat bottom plates in 180 µl TexMACS™ Medium (130-097-196, Miltenyi) and 20 µl of 10^4^ day 7 moDC cell suspension in RPMI were added in each well for a final 1:10 ratio of allogeneic DC:CD4 + naïve T cells. For the proliferation assay, CD4 + naïve T cells were labeled with CFSE (SCT110, Sigma) prior to seeding. Cells were cultured in the presence or absence of T Cell TransAct (130-111-160, Miltenyi) for the activation of T cells. After 5 days, all samples were collected and centrifuged at 300 g for 6 min. Cell pellets and supernatants were used for further analyses.

### Protein assays

SM were pooled into groups of five samples each to reach the minimum volume requirements for a custom human Quantibody array (RayBiotech). It was deemed inadvisable to dilute the samples, as this would likely result in them falling below the detection limit. Only SM from high-quality embryos (grade AA, AB, BA, or BB), graded as defined by Gardner et al., were used [26, 27]. Samples were pooled based on whether they were categorized as pregnant or non-pregnant as described in the aforementioned section on sample collection. SM were also individually analyzed with a custom human high-sensitivity ProcartaPlex 13-Plex (ThermoFisher Scientific). Day 7 moDC culture supernatants were analyzed via a commercially available Human Immune Monitoring 65-Plex ProcartaPlex (EPX650-16500-901, ThermoFisher Scientific). All assays were performed according to the manufacturer’s protocols.

### Flow cytometry

Flow cytometry (FC) analysis was performed in a DxFLEX flow cytometer (Beckman Coulter), while the collected data were analyzed with FlowJo software (BD Biosciences). Dead cells were excluded either with the fixable viability dye eFluor 780 (65-0865-14, eBioscience) or with the nucleic acid dye 7-Amino-Actinomycin D (7AAD, 559925, BD Biosciences). After a short incubation with the FcR blocking reagent (130-059-901, Miltenyi), cells were stained for 15 min with fluorophore-labeled antibody master mixes (See Supplementary Table 1, Additional File 1).

### Measurement of tryptophan, kynurenine and neopterin

Tryptophan (Trp) and its catabolite kynurenine (Kyn) were determined by HPLC as previously described [28]. Briefly, chromatographic separation was performed on an Agilent 1260 HPLC system with a LiChroCART 55 − 4 C18 column (3 μm particle size, Merck) connected to a C-18 security guard precolumn (4 mm × 3 mm, Phenomenex), using 15 mM potassium dihydrogen phosphate buffer as mobile phase, isocratic elution at a flow rate of 1.1 ml/min at 25 °C. Internal and calibration standards (Sigma Aldrich) were dissolved in aqueous albumin solution (70 g/l, AL-Labortechnik, Zeillern-Amstetten). 50 µl of the internal standard solution (3-nitro-l-tyrosine, 25 µM) was added to 50 µl of the samples or calibration standards. Proteins were precipitated with 12.5 µl trichloroacetic acid 2 M and precipitates were removed by centrifugation. Kyn and the internal standard 3-nitro-l-tyrosine were determined at a wavelength of 360 nm using a 1260 Infinity II DAD detector (G7115A, Agilent). Trp was determined via their native fluorescence using a 1260 Infinity II fluorescence detector (G7121B, Agilent) (Trp: excitation wavelength of 286 nm, emission wavelength of 366 nm). The ratio of Kyn/Trp was calculated as surrogate for IDO-1 activity [29]. Neopterin concentrations were measured by ELISA (BRAHMS Diagnostica) [30].

### Statistical analysis

For the comparison of the characteristics of patients and samples, all data were analyzed in IBM SPSS Statistics software. Normality was assessed by Kolmogorow-Smirnow normality test and Shapiro-Wilk normality test. Normally distributed data were analyzed using one-way ordinary ANOVA. Non-normally distributed data were analyzed using Kruskal-Wallis test. Qualitative outcome variables were analyzed using chi-square test or Fisher’s exact test, as appropriate. Quantitative outcome variables were presented as a mean ± SEM (standard error of mean), and qualitative outcome variables were presented as absolute number and percentage of occurrence within the group. In all cases, statistical significance was determined by a P value of less than 0.05.

Flow cytometry data were analyzed in FlowJo software (v10.8 for Windows, BD Biosciences), and the proliferation modelling tool was used for proliferation data. Multiplex data were analyzed with the Invitrogen ProcartaPlex Analysis App (ThermoFisher Scientific). A baseline corresponding to the culture medium used was removed from all protein array data. All experimental data were finally analyzed in GraphPad Prism (v10 for Windows, GraphPad Software). Normality was assessed by D’Agostino-Pearson omnibus normality test and Shapiro-Wilk normality test. Normally distributed data were analyzed using unpaired t-test or one-way ordinary ANOVA, as appropriate. Non-normally distributed data were analyzed using Mann-Whitney test or Kruskal-Wallis test, as appropriate. In all cases, statistical significance was determined by a P value of less than 0.05.

## Results

### Study population parameters

A total of 150 participants were included. The mean age was 32.07 ± 0.36 years (SD). The primary indication for ART was female infertility in 37.3% of cases. 25.3% were diagnosed with endometriosis and 14.7% with recurrent implantation failure (RIF). RIF was defined as ≥ 3 fresh or frozen blastocyst transfers with a subsequent negative serum hCG result. No statistically significant differences were found concerning age, BMI, ART diagnosis, allergies, nicotine, alcohol consumption, endometriosis, and sperm quality. No statistically significant differences were found in the hormonal profile with the exception of AMH (*p* = 0.042) and 17-β estradiol (*p* = 0.024). A statistically significant difference was found concerning RIF patients (*p* < 0.001). A total of 223 samples (*n* = 103 prSM, *n* = 120 npSM) were analyzed. No statistical significance was found concerning the method of fertilization (ICSI or IVF) and the type of embryo transfer (fresh or cryopreserved) between the prSM and npSM samples. Detailed information on patient and sample characteristics can be found in Table 1.

**Table 1:** Characteristics of patients and spent media (SM) samples

Patient Characteristics	Patients with prSM samples (*n* = 70)	Patients with prSM and npSM samples (*n* = 28)	Patients with npSM samples (*n* = 52)	*P* value
**Age** (y) ^**a**^	31.6 ± 0.5	32.1 ± 0.8	32.6 ± 0.6	0.455
**BMI** (kg/m^2^) ^**b**^				0.825
below 19	3 (4.3%)	4 (14.3%)	3 (5.8%)	
19–25	42 (60.0%)	15 (53.6%)	34 (65.4%)	
26–30	11 (15.7%)	6 (21.4%)	9 (17.3%)	
31–35	4 (5.7%)	1 (3.6%)	2 (3.8%)	
more than 35	4 (5.7%)	0 (0.0%)	1 (1.9%)	
**Diagnosis for ART** ^**b**^				0.939
Female	25 (35.7%)	11 (39.3%)	20 (38.5%)	
Male	18 (25.7%)	7 (25.0%)	10 (19.2%)	
Combined	17 (24.3%)	7 (25.0%)	12 (23.1%)	
Idiopathic	5 (7.1%)	1 (3.6%)	7 (13.5%)	
**Allergies** ^**b**^	24 (34.3%)	9 (32.1%)	16 (30.8%)	0.707
**Nicotine** (cigarettes/day) ^**b**^				0.510
0	35 (50.0%)	15 (53.6%)	30 (57.7%)	
below 5	10 (14.3%)	0 (0.0%)	6 (11.5%)	
5–10	8 (11.4%)	6 (21.4%)	5 (9.6%)	
10–20	7 (10.0%)	5 (17.9%)	5 (9.6%)	
above 20	2 (2.9%)	0 (0.0%)	1 (1.9%)	
**Alcohol** ^**b**^				0.065
no	31 (44.3%)	6 (21.4%)	27 (51.9%)	
normal consumption	16 (22.9%)	10 (35.7%)	14 (26.9%)	
above normal	0 (0.0%)	0 (0.0%)	0 (0.0%)	
**Hormonal Profile** ^**a**^				
AMH (µg/l)	4.8 ± 0.7	2.7 ± 0.6	4.0 ± 0.8	**0.042**
FSH (U/l)	7.2 ± 0.5	7.2 ± 0.5	8.1 ± 1.0	0.855
LH (U/l)	2.7 ± 0.4	2.1 ± 0.4	3.2 ± 0.6	0.701
17-β estradiol (ng/l)	1754 ± 157.6	2169 ± 197.3	1550 ± 193.7	**0.024**
Progesterone (µg/l)	1.5 ± 0.3	0.9 ± 0.1	1.3 ± 0.3	0.788
**Endometriosis** ^**b**^	16 (22.9%)	8 (28.6%)	14 (26.9%)	0.798
**RIF** ^**b**^	6 (8.6%)	7 (25.0%)	9 (17.3%)	**< 0.001**
**Sperm quality** ^**b**^				0.865
Normozoospermia	29 (41.4%)	12 (42.9%)	23 (44.2%)	
Oligozoospermia	9 (12.9%)	5 (17.9%)	3 (5.8%)	
Asthenozoospermia	5 (7.1%)	3 (10.7%)	5 (9.6%)	
Teratozoospermia	4 (5.7%)	2 (7.1%)	5 (9.6%)	
OAT	8 (11.4%)	2 (7.1%)	3 (5.8%)	
TESE	8 (11.4%)	1 (3.6%)	5 (9.6%)	
**Sample Information**	**prSM** (*n* = 103)		**npSM** (*n* = 120)	
**Embryo transfer** ^**b**^				0.467
Fresh	38 (36.9%)		50 (41.7%)	
Frozen	65 (63.1%)		70 (58.3%)	
**Method** ^**b**^				0.579
ICSI	87 (84.5%)		98 (81.7%)	
IVF	16 (15.5%)		22 (18.3%)	

Data presented as ^a^ mean ± SEM, ^b^ percentage of occurrence within the group prSM = pregnant, positive hCG 16 days post embryo transfer and detection of fetal heartbeat via ultrasound around the 7th week of pregnancy; npSM = non-pregnant, negative hCG 16 days post embryo transfer; BMI = body mass index; ART = assisted reproduction techniques; AMH = anti-muellerian hormone; FSH = follicle-stimulating hormone; LH = luteinizing Hormone; RIF = recurrent implantation failure; OAT = oligoasthenoteratospermia; TESE = testicular sperm extraction; ICSI = intracytoplasmic sperm injection; IVF = in vitro fertilization

### Preimplantation embryos secrete a variety of cytokines and growth factors

**Fig. 1:**
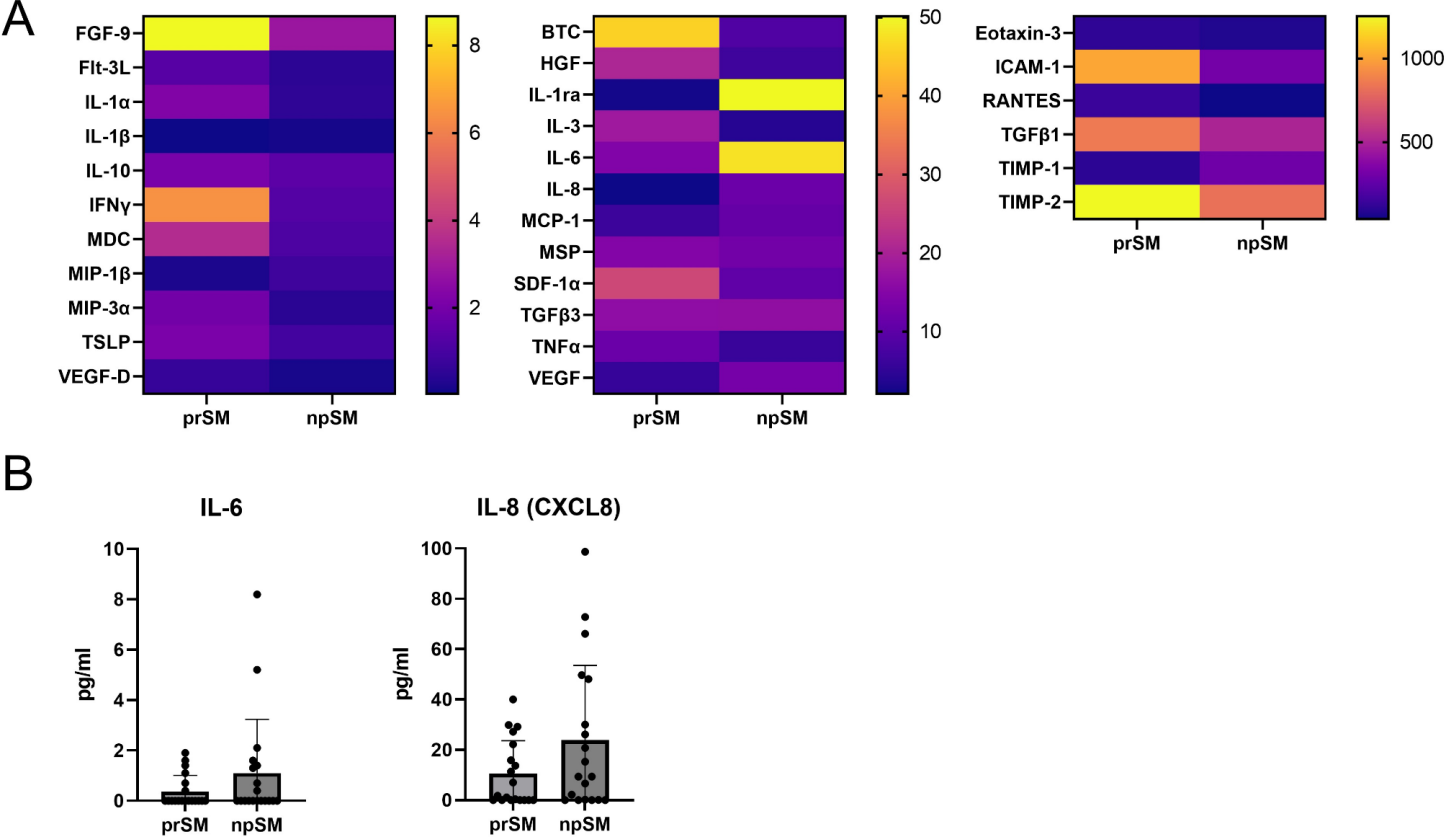
Secretion levels of cytokines in human blastocyst spent media (SM). **(A)** Custom human Quantibody protein array (RayBiotech); prSM: 15 SM samples pooled in three groups of five samples each, npSM: 20 SM samples pooled in four groups of five samples each. Baseline of embryo culture medium was removed from all samples, targets are shown grouped according to their quantity (pg/ml). **(B)** High sensitivity human ProcartaPlex protein array (Thermo Fisher Scientific); prSM *n* = 19, npSM *n* = 19. Baseline of embryo culture medium was removed from all samples. prSM = pregnant, positive hCG 16 days post embryo transfer and detection of fetal heartbeat via ultrasound around the 7th week of pregnancy; npSM = non-pregnant, negative hCG 16 days post embryo transfer

Preimplantation embryos are known to secrete a wide range of substances that are important for their development and their interaction with the maternal environment [31, 32]. To investigate potential differences in the secretion profiles between embryos that implanted and embryos that did not implant, protein analysis was conducted. Initially, pooled SM samples were analyzed using a custom human Quantibody array by RayBiotech for the detection of 40 targets. Of those, half of the targets (20/40) exhibited increased levels in prSM, while only a few (8/40) were found increased in npSM (Fig. 1A, See Supplementary Table 2, Additional File1). Eleven targets that were undetectable in all or the majority (> 50%) of the samples were excluded from further analysis. To validate the findings obtained from pooled samples, a high-sensitivity bead-based multiplex immunoassay was performed on individual samples, confirming increased secretion of IL-6 and IL-8 in npSM samples (Fig. 1B). However, GM-CSF, IFNγ, IL-1β, IL-2, IL-4, IL-5, IL-10, IL-12p70, IL-17 A, MCP-1, and TNF-α were below the detection limit in all or the majority of the samples. Based on these results, we conclude that human preimplantation embryos secrete low levels of a variety of substances in vitro. However, their detection is hindered due to the sensitivity limitations of the existing multi-target assays.

### MoDC phenotypical characterization

**Fig. 2:**
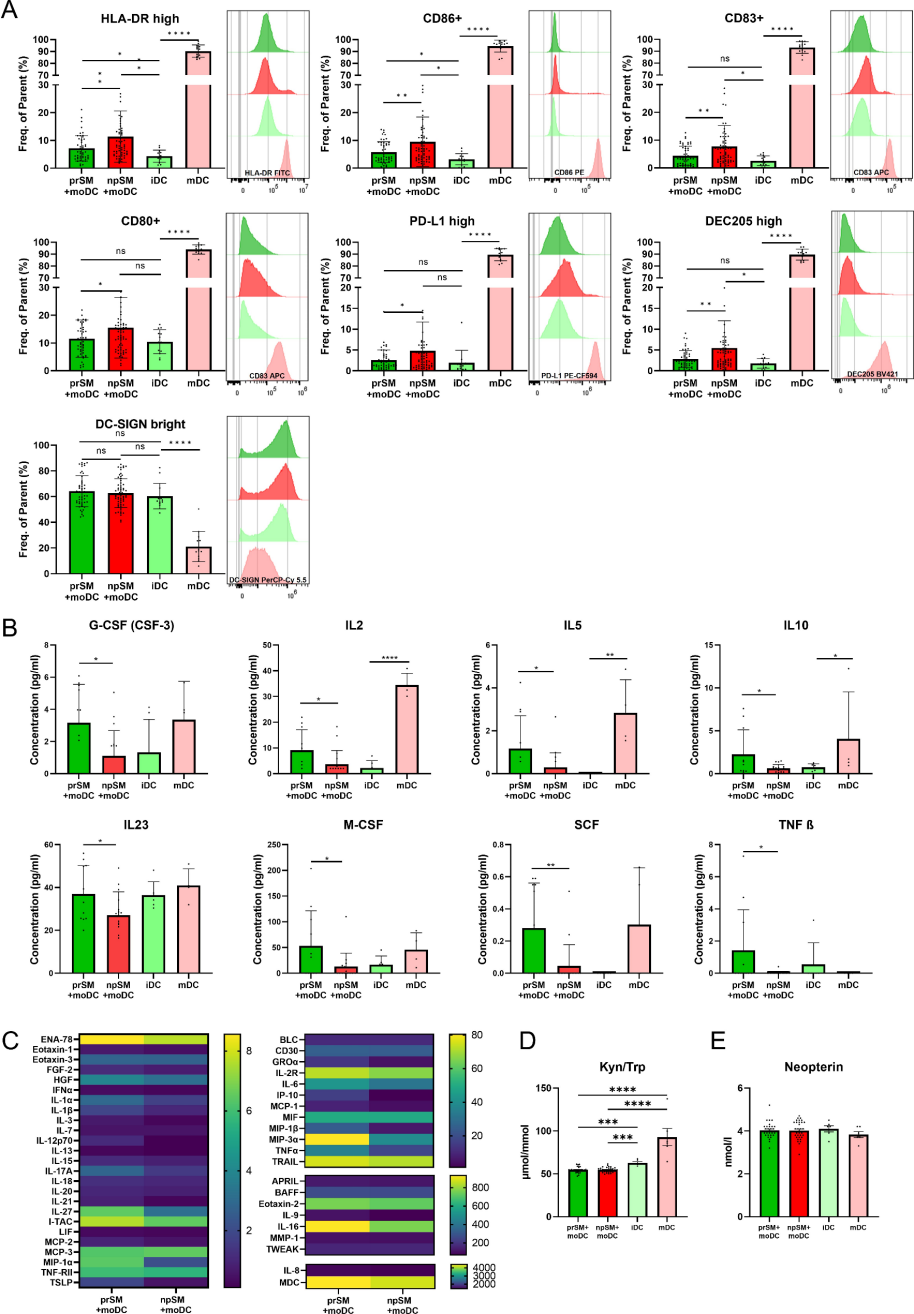
Effect of human blastocyst spent media (SM) on monocyte-derived dendritic cells (moDC). **(A)** Phenotypic characterization of moDC after stimulation with human embryo SM, graphs show the mean ± SEM (as frequency of single, live cells) and are accompanied by a representative histogram overlay of the relevant extracellular molecule, prSM + moDC *n* = 50, npSM + moDC *n* = 64, iDC *n* = 13, mDC *n* = 13; **(B)** Cytokine secretion of moDC after stimulation with human embryo SM, graphs show the mean concentration (pg/ml) ± SEM in targets with a significant difference between prSM + moDC and npSM + moDC, baseline of embryo culture medium was removed from all samples, prSM + moDC *n* = 11, npSM + moDC *n* = 17, iDC *n* = 6, mDC *n* = 4; **(C)** Detectable targets in all or most moDC samples stimulated with SM, scale represents concentration in pg/ml, baseline of embryo culture medium was removed from all samples, prSM + moDC *n* = 11, npSM + moDC *n* = 17; **(D)** IDO-1 activity as reflected by Kyn/Trp ratio, prSM + moDC *n* = 30, npSM + moDC *n* = 33, iDC *n* = 6, mDC *n* = 6; **(E)** Neopterin concentration, prSM + moDC *n* = 30, npSM + moDC *n* = 33, iDC *n* = 6, mDC *n* = 6; prSM = pregnant, positive hCG 16 days post embryo transfer and detection of fetal heartbeat via ultrasound around the 7th week of pregnancy; npSM = non-pregnant, negative hCG 16 days post embryo transfer; iDC = immature monocyte derived dendritic cells; mDC = mature monocyte derived dendritic cells. Statistical significance was determined using student t-test or Mann-Whitney test *p < 0.05; **p < 0.01; ***p < 0.001; ****p < 0.0001

To assess the immunogenic properties of human embryo SM on DC, moDC were stimulated with prSM or npSM for 48 h. The extracellular expression levels of co-stimulatory and other surface receptors and molecules were examined using flow cytometry. Stimulation of immature moDC with either prSM or npSM led to an elevated expression of all tested surface markers except for the expression of dendritic cell-specific intercellular adhesion molecule-3-grabbing non-integrin (DC-SIGN) in comparison to unstimulated moDC (iDC) (Fig. 2A).

Between the two different SM groups, stimulation of immature moDC with npSM resulted in a significant increase of the expression of all surface markers tested, except for DC-SIGN where no significant change was observed, as compared to stimulation of immature moDC with prSM. Notably, the MHC class II cell surface receptor, HLA-DR, exhibited a 59.4% (*p* = 0.0034) increase. The co-stimulatory molecules CD86 and CD80, and the maturation marker CD83, increased by 64.8% (*p* = 0,0066), 33.62% (*p* = 0,0284), and 75.5% (*p* = 0.0046), respectively. Programmed death-ligand 1 (PD-L1) was found to be expressed on the surface of all moDC groups, its expression was however further upregulated after npSM stimulation by 87.1% (*p* = 0.0314) compared to prSM stimulation. DEC205 was also found expressed in all moDC groups but like PD-L1 and HLA-DR, its expression was further upregulated after npSM stimulation by a 92.6% increase (*p* = 0.0069) compared to prSM (Fig. 2A). No significant differences were observed in the effect of SM on the moDCs in relation to RIF or endometriosis indicating that the immunogenic properties of prSM and npSM on dendritic cells were consistent, regardless of whether the embryos came from patients with RIF or endometriosis (See Supplementary Fig. 1A, Additional File 1).

### MoDC protein secretion and IDO-1 activity

To investigate the impact of human embryo SM on DC, we conducted a multiplex immunoassay for the simultaneous analysis of 65 targets. Intriguingly, distinct differences were observed in the secretory profiles of moDC stimulated with either npSM or prSM. Generally, moDC stimulation with prSM elicited a more dynamic secretory profile as compared with npSM stimulation. A significant increase was detected after stimulation with prSM in the secretion of granulocyte colony-stimulating factor (G-CSF), IL-2, IL-5, IL-10, IL-23, macrophage colony-stimulating factor (M-CSF), stem cell factor (SCF), and tumor necrosis factor beta (TNFβ) (Fig. 2B, See Supplementary Table 3, Additional File 1). Moreover, a non-statistically significant but noticeable increase (> 20%) was observed in the secretion of eotaxin-1, fibroblast growth factor 2 (FGF-2), growth-regulated alpha protein (GROα), hepatocyte growth factor (HGF), interferon alpha (IFNα), IL-1α, IL-1β, IL-6, IL-9, IL-12p70, IL-15, IL-27, interferon gamma-induced protein 10 (IP-10), LIF, monocyte chemoattractant protein-2 (MCP-2), macrophage inflammatory protein 3-alpha (MIP-3α), tumor necrosis factor alpha (TNFα), and thymic stromal lymphopoietin (TSLP). Minor or negligible differences (< 20%) were detected in the secretion levels of 21/65 analyzed targets (Fig. 2C, See Supplementary Table 3, Additional File 1). 15 targets that were undetectable in most samples (> 90%) were excluded from further analysis. IL-4 and GM-CSF were excluded from the analysis since their addition to the culture media for the differentiation of monocytes could influence the results in an unpredictable manner. Detailed concentrations are provided in Supplementary Table 3, Additional File 1. To investigate IDO-1 activity for a potential differentiation of DC under the influence of SM into a tolerogenic DC we analyzed Kyn/Trp ratio. Metabolite concentrations revealed that treatment with either prSM or npSM inhibited tryptophan breakdown to kynurenine in moDC compared to iDC and mDC, where a higher concentration of kynurenine and a higher Kyn/Trp was observed implicating a downregulation of IDO-1 activity in SM treated moDC (Fig. 2D). Concentrations of neopterin, a marker for oxidative stress and inflammation did not differ (Fig. 2E).

### SM-moDC activate T cells

**Fig. 3:**
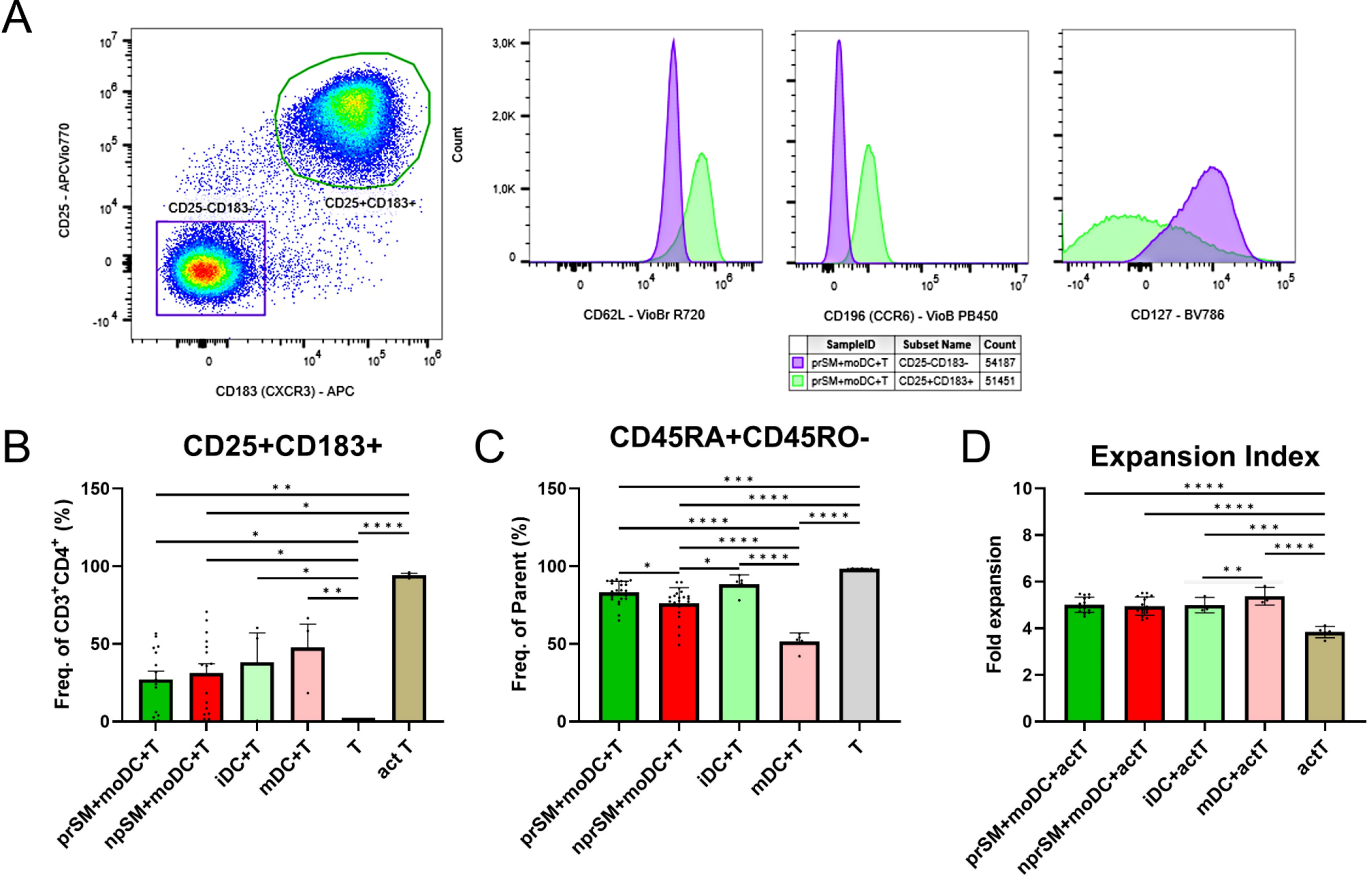
Effect of monocyte-derived dendritic cells (moDC) treated with human blastocyst spent media (SM) on naïve CD4 + T cells. **(A)** Representative density plot graph of T cell differentiation into CD25 + CD183 + and CD25-CD183- cells after culture with moDC and the expression of CD62L, CD196, and CD127 within these subpopulations (CD25 + CD183 + green; CD25-CD183- purple) as seen in histogram overlays; **(B)** Percentage of CD25 + CD183 + T cells after co-culture with moDC cells, prSM + moDC *n* = 15, npSM + moDC *n* = 16, iDC *n* = 3, mDC *n* = 3, T *n* = 3, activated T *n* = 3; **(C)** Percentage of CD45RA + CD45RO- T cells after co-culture with moDC cells, prSM + moDC *n* = 23, npSM + moDC *n* = 24, iDC *n* = 5, mDC *n* = 5, T *n* = 6; **(D)** T cell proliferation after CFSE labelling, prSM + moDC *n* = 15, npSM + moDC *n* = 16, iDC *n* = 3, mDC *n* = 3, T *n* = 5. prSM = pregnant, positive hCG 16 days post embryo transfer and detection of fetal heartbeat via ultrasound around the 7th week of pregnancy; npSM = non-pregnant, negative hCG 16 days post embryo transfer; iDC = immature monocyte derived dendritic cells; mDC = mature monocyte derived dendritic cells. Statistical significance was determined using student t-test or Mann-Whitney test *p < 0.05; **p < 0.01; ***p < 0.001; ****p < 0.0001

To evaluate the impact of SM-stimulated moDC on the activation and differentiation of T cells, CD4 + naïve T cells were co-cultured with moDC that were previously stimulated with prSM or npSM. The extracellular expression levels of specific activation cell surface markers and the proliferation of T cells were examined using flow cytometry. All moDC groups were able to induce the expression of CD25 and CD183 in T cells. CD25 is a T cell activation marker [33], while CD183 is a chemokine receptor that is upregulated on naïve T cells rapidly after their activation [34]. Moreover, these CD25 + CD183 + T cells had an elevated expression of CD62L (associated with the homing and trafficking of T cells), they were positive for CD196 (a chemokine receptor associated with Th17 cells), and negative/low for CD127 (a cell surface receptor expressed on T cells and other immune cells) when compared with the CD25-CD183- population (Fig. 3A). However, there was no significant difference in the percentage of the CD25 + CD183 + cells between the different treatments (Fig. 3B). Conversely naïve CD45RA + CD45RO- T cells were found significantly decreased after co-culture with npSM-moDC as compared to prSM-moDC (Fig. 3C). Additionally, T cells only exhibited proliferation in the co-culture when a CD3/CD28 cocktail was added but no significant differences were observed within the groups (Fig. 3D).

## Discussion

In this study, we aimed to investigate the crosstalk between maternal immune cells and the developing embryo by examining the immunogenic properties of human blastocyst spent media (SM) on dendritic cells (DC). We presented evidence by several independent experimental designs: (i) cytokine production by the embryo itself, (ii) flow cytometry analyses for phenotypical changes of SM-treated monocyte-derived DC (moDC), (iii) cytokine production of SM-treated moDC, (iv) measurement of IDO-activity of SM-treated moDC, and (v) co-culture of SM-treated moDC with naive T cells for the detection of functional changes.

Embryo SM research aims to comprehend embryo development intricacies and to pioneer non-invasive methods for embryo selection [35, 36]. Our findings demonstrate that embryos secrete in vitro a plethora of cytokines and growth factors, suggesting that embryos are able to actively interact and shape their microenvironment even before implantation. Specifically, 28 out of the 40 targets were found to be differentially expressed in pooled samples. Interestingly, 75% of the molecules found upregulated in the implanted embryo SM are part of the proinflammatory pathway while only four are part of the anti-inflammatory. This aligns with previous research showing that the first steps in human implantation require a proinflammatory environment [37–39]. IL-6 and IL-8 cytokines were detectable in embryo SM. These cytokines were previously identified in SM of blastocysts [40, 41] and have been proposed as potential markers for blastocyst grading [42–46]. However, our findings do not support the efficacy of measuring cytokines in SM for embryo grading. The limitation stems from the frequently low concentrations of individual cytokines in the culture, rendering their reliable detection challenging.

Cytokines are potent signaling molecules that can exert significant effects even at extremely low concentrations and low-grade inflammation is important for the maintenance of immune tolerance [47]. Studies have shown that the attachment of merely 4 IL-6 molecules per cell can produce statistically significant biological activity, whereas the presence of only 16 IL-6 molecules is capable of inducing substantial cellular responses [48]. Balancing cytokine production is crucial since both increases and decreases in their levels can lead to adverse outcomes [49]. For example, both elevated and reduced IL-6 concentrations have been associated with an increased risk of infertility and miscarriage [50].

Moreover, signaling molecules often exhibit pleiotropic effects and function within intricate networks. The impact of many cytokines can differ depending on their concentration and the particular tissue context [51]. Although cytokines are not currently used as markers for blastocyst grading, they may influence blastocyst implantation and early embryo development, especially in the context of the endometrial microenvironment.

Next, we found that exposure of moDC to SM led to significant alterations in their phenotype. Notably, SM from embryos that failed to implant induced an increase in the expression of co-stimulatory molecules and activation markers on moDC. These findings suggest that embryos that fail to implant may trigger a stronger response compared to those that successfully implant [1]. Studies have demonstrated that embryo rejection can elicit a more potent inflammatory immune response compared to successful implantation [52]. Moreover, excessive inflammation has been found to hinder embryo implantation, as seen in endometriosis and recurrent miscarriage patients [1, 53–55].

Furthermore, we observed that prSM-induced moDC secreted various cytokines and growth factors like G-CSF, IL-2, IL-5, and IL-10. This cytokine profile could potentially influence the immune response at the embryo-maternal interface [15]. G-CSF can enhance endometrial receptivity by promoting endometrial vascular remodeling and regulating the expression of genes associated with embryo adhesion [56]. IL-2 is part of the Th1 pro-inflammatory response that is necessary for the initial stages of implantation [57]. To allow for proper implantation while preventing excessive inflammation, a delicate balance of pro- and anti-inflammatory cytokines is maintained [47]. Both IL-5 and IL-10 are part of the Th2 anti-inflammatory response and can contribute to the establishment of an immunotolerant environment at the maternal-fetal interface [57, 58]. Interestingly, SM-treated moDC were still capable of stimulating naïve T cells, indicating that they retained their ability to activate the adaptive immune response.

IDO-1 activity as reflected by Kyn/Trp ratio was significantly downregulated in moDC after contact with SM indicating an inhibition of this specific DC function. IDO-1 inhibition may be important in the peri-implantation phase in a proinflammatory surrounding. It was shown that in DC, the expression of IDO-1 and the activation of the enzyme can be regulated independently of each other by different stimuli [59], which may not be sufficiently present in SM. Neopterin concentration did not differ between the groups. Therefore, it seems that induction of IDO-1 for tolerance may take place in another context and probably at a later time point. However further studies are needed.

Regarding the limitations of our study, there are a few important points to consider. Firstly, due to the current sensitivity restrictions of multi-targeted protein arrays, some of the SM protein array findings were obtained through pooling of SM samples. Even with a high-sensitivity protein array, many targets were below the detection limit. This was partially anticipated given the low volume and concentration of the embryo SM. In vivo, such low levels of secretion may be sufficient to affect the immediate environment of the embryo in the endometrial milieu. As technology advances, more sensitive arrays are expected. Secondly, the study included participants with varying infertility etiologies. While no statistically significant differences were observed in relation to most of the patient characteristics (the exception was AMH and 17-β estradiol levels, and RIF), we cannot determine whether SM from different infertility etiologies have distinct effects on DC within the scope of this study. A subgroup analysis of RIF and endometriosis patients revealed no significant differences regarding the effect of SM on the phenotype of moDC. However, due to the small number of RIF and endometriosis patients, a separate comparison in all the experimental settings was not possible. A future study focused on RIF patients could provide valuable insights. Thirdly, due to the scarcity of endometrial DC, we resorted to using moDC isolated from the peripheral blood of healthy women, a commonly substituted alternative. Although this was an unavoidable limitation, we cannot determine whether endometrial DC, which have been shown to have unique characteristics [60], would react the same way as moDC when treated with SM. Furthermore, the blastocysts that were included in the study were not subjected to genetic screening prior to implantation. This was in accordance with national regulations, however, ensuring that all the embryos included in the study were euploid could have resulted in a more homogeneous study population. Lastly, our experimental setup represents an artificial in vitro environment due to the unavailability of alternative options. Despite these constraints, our study offers valuable insights into maternal immune tolerance and presents new avenues for further research.

## Conclusion

In conclusion, our study reveals that human preimplantation embryos secrete a wide range of immunomodulatory molecules that significantly affected the phenotype and function of monocyte derived dendritic cells. Understanding the immunomodulatory properties of preimplantation embryos and their effects on maternal immune cells may prevent pregnancy complications associated with immune dysregulation and enhance implantation success rates.

## Electronic supplementary material

Below is the link to the electronic supplementary material.

Supplementary Material 1

## Data Availability

The datasets used and/or analysed during the current study are available from the corresponding author on reasonable request.

## Abbreviations

AMH: Anti-Müllerian Hormone
APC: Antigen Presenting Cells
APLS: Antiphospholipid Syndrome
ART: Assisted Reproductive Technology
CFSE: Carboxyfluorescein Succinimidyl Ester
CSCM-NX: Continuous Single Culture-NX
DC: Dendritic Cells
FBS: Fetal Bovine Serum
FC: Flow Cytometry
FGF-2: Fibroblast Growth Factor 2
G-CSF: Granulocyte Colony-Stimulating Factor
GM-CSF: Granulocyte-Macrophage Colony-Stimulating Factor
GROα: Growth-Regulated Alpha Protein
Hcg: Human Chorionic Gonadotropin
HEPES: 4-(2-hydroxyethyl)-1-piperazineethanesulfonic acid
HGF: Hepatocyte Growth Factor
HLA-DR: Human Leucocyte Antigen DR
ICSI: Intracytoplasmic Sperm Injection
IDO-1: Indoleamine 2,3-dioxygenase 1
IFNα: Interferon Alpha
IL1β: Interleukin 1β
IP-10: Interferon gamma-induced Protein 10
IVF: In Vitro Fertilization
Kyn: Kynurenine
LIF: Leukemia Inhibitory Factor
LRSC: Leucocyte Reduction System Chambers
MCP-2: Monocyte Chemoattractant Protein-2
M-CSF: Macrophage Colony-Stimulating Factor
MIP-3α: Macrophage Inflammatory Protein 3-alpha
moDC: Monocyte Derived Dendritic Cells
NK: Natural Killer
npSM: Non-pregnant Spent Media
PBMC: Peripheral Blood Mononuclear Cells
PD-1: Programmed Death-1
PGE2: Prostaglandin E2
prSM: Pregnant Spent Media
RIF: Recurrent Implantation Failure
RPMI: Roswell Park Memorial Institute Medium
SCF: Stem Cell Factor
SEM: Standard Error of Mean
sFlt1: soluble Fms-like tyrosine kinase-1
SM: Spent Media
SSS: Serum Substitute Supplement
TGF-β1: Transforming Growth Factor β1
Tim-3: T-cell immunoglobulin mucin-3
TNFα: Tumor Necrosis Factorα
TNFβ: Tumor Necrosis Factor Beta
Trp: Tryptophan
TSLP: Thymic Stromal Lymphopoietin
