# Supplementary material for: Dendritic cells under the control of the preimplantation embryo secretome: an in vitro study
Source: Reprod Biol Endocrinol. 2024 Nov 23;22:150. doi: 10.1186/s12958-024-01319-2 (PMC11585248; doi:10.1186/s12958-024-01319-2)
Supplement: Supplementary file 1 — Supplementary Material 1 [file 12958_2024_1319_MOESM1_ESM.docx]

# Supplementary


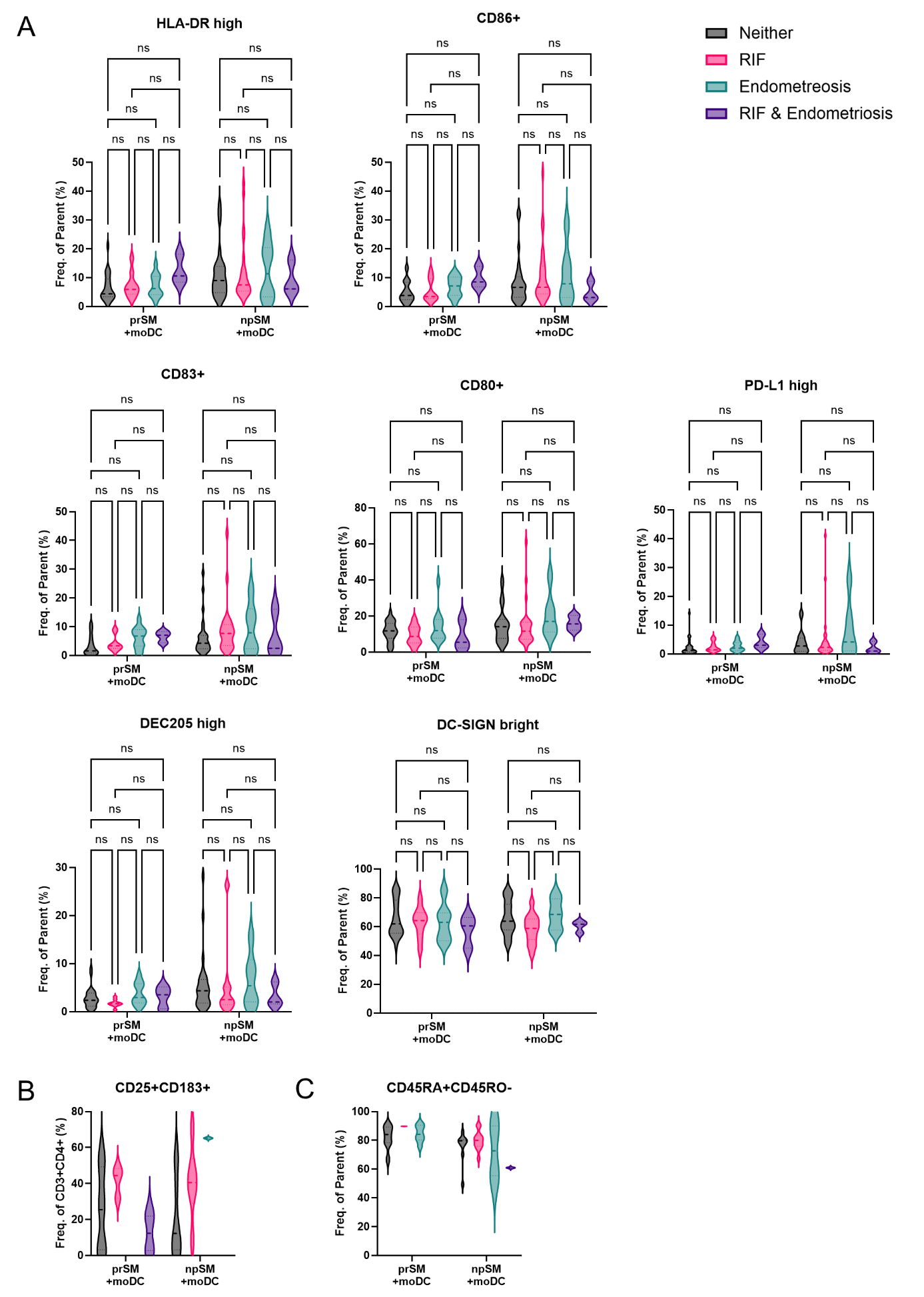


**Supplementary Figure 1. Comparison of SM samples from patients with or without RIF and endometriosis**

**A.** Phenotypic characterization of moDC after stimulation with human embryo SM (as frequency of single, live cells) accompanied by a representative histogram overlay of the relevant extracellular molecule, prSM from patients with neither RIF nor endometriosis + moDC n=26, prSM from patients with RIF + moDC n=9, prSM from patients with endometriosis + moDC n=12, prSM from patients with both RIF and endometriosis + moDC n=3; npSM from patients with neither RIF nor endometriosis + moDC n=28, npSM from patients with RIF + moDC n=17, npSM from patients with endometriosis + moDC n=6, prSM from patients with both RIF and endometriosis + moDC n=3.

**B.** Percentage of CD25+CD183+ T cells after co-culture with moDC cells, prSM from patients with neither RIF nor endometriosis + moDC n=10, prSM from patients with RIF + moDC n=3, prSM from patients with endometriosis + moDC n=0, prSM from patients with both RIF and endometriosis + moDC n=2; npSM from patients with neither RIF nor endometriosis + moDC n=9, npSM from patients with RIF + moDC n=6, npSM from patients with endometriosis + moDC n=1, prSM from patients with both RIF and endometriosis + moDC n=0.

**C.** Percentage of CD45RA+CD45RO- T cells after co-culture with moDC cells, prSM from patients with neither RIF nor endometriosis + moDC n=14, prSM from patients with RIF + moDC n=2, prSM from patients with endometriosis + moDC n=7, prSM from patients with both RIF and endometriosis + moDC n=0; npSM from patients with neither RIF nor endometriosis + moDC n=9, npSM from patients with RIF + moDC n=12, npSM from patients with endometriosis + moDC n=2, prSM from patients with both RIF and endometriosis + moDC n=1.

Supplementary Table 1. Antibody Information.

| **Target** | **Epitope** | **Company** | **Panel** |
| --- | --- | --- | --- |
| *CD14* | HCD14 | BD Biosciences | Monocytes |
| *CD40* | 5C3 | BD Biosciences | DC |
| *CD80* | L307.4 | BD Biosciences | DC |
| *CD83* | HB15e | BD Biosciences | DC |
| *CD86* | IT2.2 | BD Biosciences | DC |
| *CD205* | MMRI-7 | BD Biosciences | DC |
| *CD209* | DCN46 | BD Biosciences | DC |
| *CD274* | MIH1 | BD Biosciences | DC |
| *HLA-DR* | G46-6 | BD Biosciences | DC |
| *CCR10* | REA326 | Miltenyi | T cell 1 |
| *CD3* | REA613 | Miltenyi | T cell 1 |
| *CD4* | REA623 | Miltenyi | T cell 1 |
| *CD25* | REA570 | Miltenyi | T cell 1 |
| *CD62L* | REA615 | Miltenyi | T cell 1 |
| *CD183* | REA232 | Miltenyi | T cell 1 |
| *CD194* | REA279 | Miltenyi | T cell 1 |
| *CD196* | REA190 | Miltenyi | T cell 1 |
| *CD127* | HIL-7R-M21 | BD Biosciences | T cell 1 |
| *CD3* | SK7 | BD Biosciences | T cell 2, CD4+ naïve |
| *CD4* | SK3 | BD Biosciences | T cell 2, CD4+ naïve |
| *CD8* | SK1 | BD Biosciences | CD4+ naïve |
| *CD45* | 2D1 | BD Biosciences | T cell 2, CD4+ naïve |
| *CD45RA* | HI100 | BD Biosciences | T cell 2, CD4+ naïve |
| *CD45RO* | UCHL1 | BD Biosciences | T cell 2, CD4+ naïve |
| *CD62L* | DREG-56 | BD Biosciences | T cell 2 |
| *CD197* | 150503 | BD Biosciences | T cell 2 |
| *7AAD* | - | BD Biosciences | Live/Dead |
| *e780* | - | eBiosciences | Live/Dead |

Monocyte derived dendritic cells (moDC) were stained with CD40, CD80, CD83, CD86, CD205, CD209, CD274, and HLA-DR (BD Biosciences), while T cells were stained either with CCR10, CD3, CD4, CD25, CD62L, CD183, CD194, CD196 (Miltenyi) and CD127 (BD Biosciences) or with CD3, CD4, CD45, CD45RA, CD45RO, CD62L, CD197 (BD Biosciences)

Supplementary Table 2. Secretion levels of cytokines in human embryo spent media.

| **Target** | **prSM ^a^ (pg/ml)** | **npSM ^a^ (pg/ml)** | **prSM ^b^ above detection** | **npSM ^b^ above detection** |
| --- | --- | --- | --- | --- |
| ***BTC*** | 45.7 | 8.5 | 100% | 50% |
| ***Eotaxin-3*** | 105.7 | 74.9 | 66.6% | 100% |
| ***FGF-9*** | 8.7 | 2.9 | 100% | 50% |
| ***Flt-3L*** | 1.3 | 0.5 | 100% | 100% |
| ***HGF*** | 20.5 | 6.4 | 100% | 25% |
| ***ICAM-1*** | 1006.6 | 309.8 | 100% | 100% |
| ***IFNγ*** | 6.4 | 1.3 | 66.6% | 25% |
| ***IL-1α*** | 2.3 | 0.5 | 100% | 50% |
| ***IL-1β*** | 0.0 | 0.1 | 33.3% | 100% |
| ***IL-1ra*** | 2.5 | 50.1 | 66.6% | 75% |
| ***IL-3*** | 18.3 | 3.8 | 66.6% | 75% |
| ***IL-6*** | 14.3 | 47.5 | 66.6% | 100% |
| ***IL-8*** | 2.1 | 11.7 | 66.6% | 100% |
| ***IL-10*** | 2.1 | 1.5 | 100% | 75% |
| ***MCP-1*** | 6.2 | 10.9 | 100% | 100% |
| ***MDC*** | 3.5 | 1.1 | 100% | 75% |
| ***MIP-1β*** | 0.2 | 0.8 | 66.6% | 75% |
| ***MIP-3α*** | 1.9 | 0.4 | 66.6% | 75% |
| ***MSP*** | 14.7 | 12.7 | 100% | 100% |
| ***RANTES*** | 139.3 | 41.5 | 100% | 100% |
| ***SDF-1a*** | 26.4 | 10.2 | 66.6% | 50% |
| ***TGFβ1*** | 849.1 | 497.8 | 100% | 25% |
| ***TGFβ3*** | 16.2 | 16.5 | 100% | 100% |
| ***TIMP-1*** | 101.9 | 295.9 | 100% | 100% |
| ***TIMP-2*** | 1251.6 | 816.5 | 100% | 100% |
| ***TNFα*** | 11.4 | 5.9 | 100% | 100% |
| ***TSLP*** | 2.1 | 0.9 | 66.6% | 75% |
| ***VEGF*** | 5.6 | 13.1 | 66.6% | 75% |
| ***VEGF-D*** | 0.7 | 0.2 | 66.6% | 50% |

Custom human Quantibody protein array (RayBiotech); prSM: n= 15 SM, where SM samples were pooled in three groups of five SM samples each, npSM: n= 20 SM, where SM samples were pooled in four groups of five SM samples each. Baseline of embryo culture medium removed from all samples. Data presented as ^a^ mean of pooled samples (average sum per batch), ^b^ percentage of samples above the detection limit within the group. prSM = pregnant, positive hCG 16 days post embryo transfer and detection of fetal heartbeat via ultrasound around the 7^th^ week of pregnancy; npSM = non-pregnant, negative hCG 16 days post embryo transfer; BTC = betacellulin; FGF-9 = fibroblast growth factor 9; Flt-3L = fms-related tyrosine kinase 3 ligand; HGF = hepatocyte growth factor; ICAM-1 = intercellular adhesion molecule 1; IFNγ = interferon gamma; MCP-1 = monocyte chemoattractant protein-1; MDC = macrophage-derived chemokine; MIP-1β = macrophage inflammatory protein 1-beta; MIP-3α= macrophage inflammatory protein 3-alpha; MSP = macrophage stimulating protein; RANTES = regulated on activation, normal T cell expressed and secreted; SDF-1α = stromal cell-derived factor 1 alpha; TGFβ1 = transforming growth factor beta-1; TGFβ3 = transforming growth factor beta-3; TIMP-1 = tissue inhibitors of metalloproteinase 1; TIMP-2 = tissue inhibitors of metalloproteinase 2; TNFα = tumor necrosis factor alpha; TSLP = thymic stromal lymphopoietin; VEGF = vascular endothelial growth factor; VEGF-D = vascular endothelial growth factor D.

Supplementary Table 3. Cytokine secretion of moDC stimulated with human embryo SM.

| Target | prSM-moDC ^a^ (pg/ml) | npSM-moDC ^a^ (pg/ml) | prSM-moDC ^b^ above detection | npSM-moDC ^b^ above detection | *p* value | iDC | mDC |
| --- | --- | --- | --- | --- | --- | --- | --- |
| APRIL | 127.0 ± 28.7 | 116.8 ± 28.5 | 100% | 100% | 0.364 | 130.8 ± 48.5 | 98.8 ± 27.6 |
| BAFF | 264.3 ± 39.3 | 262.3 ± 39.1 | 100% | 100% | 0.896 | 246.4 ± 23.6 | 256.0 ± 35.8 |
| BLC | 10.5 ± 7.7 | 10.5 ± 6.8 | 73% | 82% | 0.992 | 9.6 ± 5.7 | 2.4 ± 4.7 |
| CD30 | 24.8 ± 3.7 | 24.1 ± 5.3 | 100% | 100% | 0.691 | 23.2 ± 3.4 | 27.2 ± 3.1 |
| ENA-78 | 8.6 ± 4.5 | 7.7 ± 5.0 | 100% | 100% | 0.655 | 7.5 ± 3.5 | 10.6 ± 7.4 |
| Eotaxin-1 | 0.6 ± 0.5 | 0.4 ± 0.6 | 82% | 71% | 0.376 | 0.2 ± 0.2 | 2.8 ± 0.7 |
| Eotaxin-2 | 755.2 ± 267.1 | 724.5 ± 257.1 | 100% | 100% | 0.764 | 884.7 ± 485.3 | 329.3 ± 88.1 |
| Eotaxin-3 | 2.8 ± 0.8 | 2.7 ± 1.0 | 100% | 100% | 0.877 | 3.1 ± 1.1 | 1.7 ± 0.9 |
| FGF-2 | 1.1 ± 1.1 | 0.7 ± 0.8 | 55% | 47% | 0.227 | 1.2 ± 1.5 | 0.0 ± 0.0 |
| G-CSF | 3.2 ± 2.4 | 1.1 ± 1.6 | 73% | 41% | **0.010** | 1.3 ± 2.1 | 3.4 ± 2.4 |
| GROα | 13.5 ± 18.5 | 4.6 ± 2.2 | 100% | 100% | 0.059 | 5.0 ± 1.5 | 9.8 ± 1.4 |
| HGF | 3.9 ± 2.6 | 3.1 ± 3.3 | 100% | 94% | 0.494 | 3.0 ± 0.8 | 96.6 ± 46.6 |
| IFNα | 0.3 ± 0.3 | 0.2 ± 0.2 | 82% | 71% | 0.207 | 0.3 ± 0.2 | 0.6 ± 0.1 |
| IL-10 | 2.3 ± 2.8 | 0.6 ± 0.5 | 91% | 94% | **0.026** | 0.8 ± 0.4 | 4.1 ± 5.5 |
| IL-12p70 | 1.1 ± 2.0 | 0.1 ± 0.2 | 64% | 53% | 0.052 | 0.0 ± 0.1 | 1.1 ± 1.3 |
| IL-13 | 0.2 ± 0.4 | 0.1 ± 0.2 | 36% | 18% | 0.183 | 0.0 ± 0.0 | 1.7 ± 1.1 |
| IL-15 | 1.1 ± 0.9 | 0.8 ± 0.9 | 91% | 82% | 0.352 | 0.5 ± 0.3 | 3.2 ± 0.8 |
| IL-16 | 932.3 ± 395.3 | 760.0 ± 418.9 | 100% | 100% | 0.287 | 1156.6 ± 417.9 | 1177.9 ± 308.4 |
| IL-17A | 2.8 ± 3.5 | 1.5 ± 3.0 | 55% | 35% | 0.296 | 0.0 ± 0.0 | 4.7 ± 3.7 |
| IL-18 | 1.2 ± 1.0 | 1.0 ± 0.9 | 91% | 88% | 0.565 | 0.6 ± 0.3 | 2.2 ± 0.6 |
| IL-1α | 2.9 ± 2.9 | 1.7 ± 1.2 | 100% | 100% | 0.135 | 1.5 ± 1.2 | 5.6 ± 0.3 |
| IL-1β | 1.8 ± 1.4 | 1.0 ± 1.4 | 82% | 47% | 0.143 | 0.8 ± 1.3 | 5009.6 ± 537.3 |
| IL-2 | 9.1 ± 8.0 | 3.6 ± 5.3 | 82% | 65% | **0.038** | 2.2 ± 2.8 | 34.4 ± 4.6 |
| IL-20 | 1.0 ± 0.8 | 0.8 ± 0.7 | 73% | 76% | 0.552 | 0.9 ± 0.8 | 0.1 ± 0.2 |
| IL-21 | 0.8 ± 1.1 | 0.2 ± 0.7 | 55% | 24% | 0.078 | 0.0 ± 0.1 | 11.5 ± 1.8 |
| IL-23 | 36.9 ± 13.2 | 27.0 ± 10.9 | 100% | 94% | **0.041** | 36.4 ± 6.3 | 40.9 ± 7.8 |
| IL-27 | 6.5 ± 7.5 | 3.1 ± 5.9 | 82% | 53% | 0.196 | 2.4 ± 3.1 | 17.1 ± 3.9 |
| IL-2R | 72.3 ± 17.9 | 66.4 ± 22.2 | 100% | 100% | 0.466 | 62.3 ± 20.2 | 3754.9 ± 577.6 |
| IL-3 | 0.7 ± 1.4 | 0.2 ± 0.9 | 36% | 6% | 0.327 | 0.0 ± 0.0 | 0.0 ± 0.0 |
| IL-5 | 1.2 ± 1.5 | 0.3 ± 0.7 | 55% | 24% | **0.046** | 0.0 ± 0.0 | 2.8 ± 1.5 |
| IL-6 | 42.3 ± 24.1 | 31.8 ± 24.2 | 100% | 100% | 0.271 | 27.3 ± 17.7 | 1916.1 ± 162.7 |
| IL-7 | 0.5 ± 0.1 | 0.5 ± 0.1 | 100% | 100% | 0.358 | 0.6 ± 0.3 | 0.3 ± 0.0 |
| IL-8 | 1464.2 ± 1232.9 | 1438.2 ± 1404.3 | 100% | 100% | 0.960 | 1215.6 ± 1185.8 | 1618.7 ± 1079.1 |
| IL-9 | 105.4 ± 98.2 | 69.6 ± 45.5 | 100% | 100% | 0.201 | 68.7 ± 54.9 | 251.8 ± 22.5 |
| IP-10 | 15.9 ± 46.7 | 1.3 ± 1.3 | 64% | 65% | 0.205 | 2.2 ± 0.5 | 4.1 ± 0.9 |
| I-TAC | 7.6 ± 5.8 | 6.5 ± 5.2 | 91% | 76% | 0.602 | 7.9 ± 6.7 | 1.6 ± 3.2 |
| LIF | 0.3 ± 0.3 | 0.2 ± 0.4 | 64% | 47% | 0.593 | 0.0 ± 0.0 | 2.2 ± 0.8 |
| MCP-1 | 8.5 ± 25.1 | 4.0 ± 11.7 | 36% | 18% | 0.527 | 2.2 ± 5.4 | 1.9 ± 2.7 |
| MCP-2 | 0.9 ± 1.1 | 0.5 ± 0.2 | 100% | 100% | 0.171 | 0.5 ± 0.2 | 0.5 ± 0.1 |
| MCP-3 | 6.3 ± 2.0 | 6.5 ± 2.6 | 100% | 100% | 0.817 | 6.5 ± 2.5 | 9.2 ± 2.6 |
| M-CSF | 53.0 ± 68.2 | 12.8 ± 26.3 | 55% | 53% | **0.036** | 16.8 ± 16.6 | 46.0 ± 32.6 |
| MDC | 4416.2 ± 2386.7 | 4209.2 ± 2051.2 | 100% | 100% | 0.809 | 5452.1 ± 3634.8 | 1323.8 ± 409.6 |
| MIP-1α | 6.4 ± 12.6 | 2.1 ± 7.9 | 27% | 12% | 0.277 | 0.0 ± 0.0 | 0.0 ± 0.0 |
| MIP-1β | 23.7 ± 46.7 | 6.1 ± 12.7 | 36% | 35% | 0.149 | 0.4 ± 0.9 | 0.9 ± 1.4 |
| MIP-3α | 80.5 ± 102.7 | 38.6 ± 37.2 | 91% | 94% | 0.134 | 21.4 ± 17.9 | 70.4 ± 34.0 |
| MMP-1 | 104.3 ± 137.6 | 101.1 ± 112.6 | 100% | 100% | 0.947 | 64.1 ± 132.1 | 67.7 ± 112.4 |
| SCF | 0.3 ± 0.3 | 0.0 ± 0.1 | 55% | 12% | **0.006** | 0.0 ± 0.0 | 0.3 ± 0.4 |
| TNF-RII | 5.9 ± 4.6 | 5.6 ± 4.3 | 82% | 82% | 0.854 | 6.3 ± 5.2 | 3.5 ± 4.8 |
| TNFα | 35.2 ± 42.3 | 17.8 ± 12.8 | 100% | 100% | 0.123 | 14.2 ± 4.1 | 790.9 ± 117.9 |
| TNFβ | 1.4 ± 2.5 | 0.0 ± 0.1 | 36% | 6% | **0.028** | 0.6 ± 1.3 | 0.0 ± 0.0 |
| TRAIL | 75.2 ± 20.0 | 71.7 ± 21.1 | 100% | 100% | 0.665 | 70.6 ± 21.3 | 52.6 ± 11.9 |
| TSLP | 1.9 ± 2.8 | 0.5 ± 1.1 | 64% | 29% | 0.092 | 0.0 ± 0.1 | 0.0 ± 0.0 |
| TWEAK | 157.2 ± 68.1 | 158.1 ± 82.8 | 100% | 100% | 0.975 | 157.5 ± 90.9 | 187.0 ± 54.0 |

prSM+MoDC n=11, npSM+MoDC n=17, iDC n=6, mDC n=4. Baseline of culture medium removed from all samples. Data presented as ^a^ mean ± SD, ^b^ percentage of samples above the detection limit within the group. prSM = pregnant, positive hCG 16 days post embryo transfer and detection of fetal heartbeat via ultrasound around the 7^th^ week of pregnancy; npSM = non-pregnant, negative hCG 16 days post embryo transfer; moDC = monocyte derived dendritic cells; iDC = immature monocyte derived dendritic cells; mDC = mature monocyte derived dendritic cells.
